# Development of the social perception pathway across the third trimester of gestation and perinatal transition

**DOI:** 10.1162/IMAG.a.1394

**Published:** 2026-10-15

**Authors:** Katarzyna Chawarska, Dustin Scheinost, R. Todd Constable, Cheryl Lacadie, Huili Sun, Joseph Chang, Laura R. Ment

**Affiliations:** Child Study Center, Yale School of Medicine, New Haven, CT, United States; Department of Statistics and Data Science, Yale University, New Haven, CT, United States; Department of Radiology and Biomedical Engineering, Yale School of Medicine, New Haven, CT, United States; Departments of Pediatrics and Neurology, Yale School of Medicine, New Haven, CT, United States

**Keywords:** fetuses, neonates, functional connectivity, ventral pathway, social perception pathway

## Abstract

Babies display social perceptual skills within hours of birth which may indicate that the neural predecessors of such skills have prenatal origins. However, the putative fetal neural infrastructure supporting these skills remains unexplored. Data from 497 participants scanned between 28–44 weeks postmenstrual age were examined to map the development of intrinsic functional connectivity (iFC) within the proto social pathway (pSP), encompassing the superior temporal sulcus (STS), a region implicated in processing multimodal social stimuli later in development, and the proto ventral pathway (pVP), including the fusiform face area (FFA), a region associated with static face processing in older children and adults. IFC was greater than 0 in the bilateral pSP and pVP by 28 weeks and increased significantly in the left and right pSP and left pVP but not in the right pVP between 28 and 44 weeks. By 44 weeks, both pathways exhibited robust right-dominance. By 40 weeks, iFC for the right and left pSP was significantly higher than in the corresponding pVPs. Lastly, the right posterior STS (pSTS) and the right FFA coupling became positive by 40 and 44 weeks and the left pSTS-FFA coupling became negative by 44 weeks. These findings demonstrate that neural architecture supporting social perception later in life is present in the third trimester and undergoes rapid development across the pre- to postnatal transition. The strengthening of intrinsic connectivity, emergence of right-hemisphere dominance, and early differentiation of putative social perception and face-processing circuitry indicate that key features of the social brain begin to assemble before birth, providing a developmental scaffold for later social development.

## 1 Introduction

Emerging data suggest fetuses are social beings. In the early weeks of the third trimester of pregnancy, they respond to sound (Johansson et al., 1992), increase their heart rates to their mother’s voices (Kisilevsky & Hains, 2011), orient their heads toward upright but not inverted face-like light displays (Reid et al., 2017), touch the uterine wall in response to maternal touch (Marx & Nagy, 2017), and respond to a language learning task (Partanen et al., 2013). Furthermore, in the hours and days after birth newborns orient preferentially to face-like stimuli (Frantz, 1963; Valenza et al., 1996), to speech rather than non-speech sounds (Issard et al., 2023; Vouloumanos & Werker, 2007), to faces with direct rather than averted gaze (Farroni et al., 2002, 2006), and form elementary intermodal pairings of faces, gaze direction, and voices (Coulon et al., 2011; Guellai & Streri, 2011; Sai, 2005). Together, these behaviors suggest that proto neural pathways supporting processing of social stimuli may be present before birth, but the putative neural infrastructure supporting these pathways during the fetal and neonatal periods remains largely unexplored.

The recently described right-lateralized social perception pathway (SP) is thought to subserve the dynamic aspects of human social perception (Pitcher & Ungerleider, 2021). The proto social pathway (pSP) comprised neuroanatomical and functional inputs from the visual cortex to the motion-selective middle temporal area and into the superior temporal sulcus (STS) in the human brain (Pitcher et al., 2014, 2020). One of the first sulci to develop in the fetal brain (Habas et al., 2012), the STS supports emerging social perception (Jardri et al., 2008, 2012; Moser et al., 2021). While the right STS is implicated in dynamic aspects of social perception including faces, gaze, speech, and their combinations later in development (Lahnakoski et al., 2012; Young et al., 2020), the left STS is primarily involved in processing speech and multimodal integration of speech and sematic concepts in older children and adults (Kausel et al., 2024; Liebenthal et al., 2014; Michon et al., 2022; Specht & Reul, 2003). In term neonates, intrinsic functional connectivity (iFC) is > 0 for both the left and right SP. Notably, iFC in the right but not the left pSP pathway at birth is positively correlated with attention to dynamic socially engaging faces at age 4 months, which, in turn, associates with better social outcomes during early childhood (Chawarska et al., 2025). Relatedly, a disruption in iFC in the right pSP has been reported in children with autism spectrum disorder (Li et al., 2024).

Here, we contrast the development of the pSP pathway with the proto ventral pathway (pVP), which extends from the primary visual cortex through the lateral occipital complex and into the fusiform gyrus (FFG) (Duchaine & Yovel, 2015; Gschwind et al., 2012). In adults, the fusiform face area (FFA) of the inferior mid-FFG is involved in the processing of invariant features in static faces, including their identity and expression (Fox et al., 2009; Haxby et al., 2001; Peelen et al., 2009; Pitcher et al., 2011), and identification of familiar voices (Rhone et al., 2023; Schall et al., 2013; von Kriegstein et al., 2005). Although the pVP is bilateral (Corbetta et al., 2008), similar to the pSP, face processing is largely right-lateralized in older infants, children, and adults (Buiatti et al., 2019; Lesinger et al., 2023; Rossion & Lochy, 2022). Positive iFC between the nodes in the pVP has been previously reported in full-term neonates, suggesting this is an early maturing pathway (Chawarska et al., 2025; Kamps et al., 2020). However, unlike the right pSP, iFC levels within the ventral pathway were not associated with social attention at age 4 months (Chawarska et al., 2025).

Age-related changes in iFC across the third trimester of gestation have been shown in numerous networks in the developing brain (Ji et al., 2024; Thomason et al., 2014). In the present study, we examined the development of the pSP and pVP in the left and right hemispheres across the third trimester and perinatal transition in a large cohort of fetuses and neonates from the Developing Human Connectome Project (dHCP) (Edwards et al., 2022; Fitzgibbon et al., 2020; Karolis et al., 2025). It is not known if—as in other cortical networks, such as the default mode and salience networks (Scheinost et al., 2024; Turk et al., 2019)—iFC within the pSP and pVP is weaker before birth and strengthens only during the postnatal period. Alternatively, as reported for iFC from the posterior cingulate cortex to both the dorsal attention and executive control networks, these pathways may already show positive iFC across the third trimester of gestation (Thomason et al., 2014, 2015).

In addition, the face-specific region of the right FFG, and the right posterior STS (pSTS), the face-specific region of the STS (Isik et al., 2017), show positive iFC in adults (Turk-Browne et al., 2010). Thus, in a secondary analysis we examined connectivity between these proto nodes across the same time interval. Positive intra-hemispheric iFC between these nodes may suggest emergence of nascent functional architecture that is known to support social cognition later in life.

To the best of our knowledge, the development of the functional neural infrastructure of putative social and face perception pathways has not yet been studied during the pre- to postnatal transition.

## 2 Methods

Participants were recruited as part of the developing Human Connectome Project (dHCP), a cross-sectional Open Science initiative studying fetal and infant brain development that was approved by the UK National Research Ethics Authority (14/LO/1169) and funded by the European Research Council. Written informed consent was obtained by all participating families prior to imaging.

### 2.1 dHCP fetal sample

The initial dHCP fetal sample included 167 fetuses of 27.5 to 38.29 weeks post menstrual age (PMA). The 27.5 PMA lower cutoff was employed to accommodate the age below which our registration algorithms could not account for the lack of folding around the developing insula. Pregnant persons were prospectively recruited as part of the dHCP study, and underwent MRI studies at St. Thomas’ Hospital, London. Inclusion criteria for fetuses were (1) singleton pregnancy; (2) term birth (i.e., ≥ 37 weeks PMA); (3) birth weight appropriate for PMA (CDC, 2025); and (4) no reported cerebral structural changes. Brains of all fetuses were reviewed by a neuroradiologist as showing appropriate appearances on the T2-weighted anatomical scan for their PMA with no acquired lesions or congenital malformations of clinical significance (Karolis et al., 2025).

### 2.2 dHCP neonatal sample

The initial dHCP sample included 800 full-term neonates. Infants were recruited from the postnatal wards at St Thomas’ Hospital, London, and imaged at 37.4 to 44.9 weeks PMA at the Evelina Newborn Imaging Centre, Centre for the Developing Brain, King’s College London.

Inclusion criteria for neonates were identical to those in the fetal sample: (1) singleton pregnancy; (2) term birth (ie, ≥37 weeks PMA); (3) birth weight appropriate for PMA (CDC, 2025); and (4) no reported cerebral structural changes (Edwards et al., 2022).

### 2.3 Imaging acquisition

#### 2.3.1 dHCP fetal studies

Resting-state fMRI data were acquired with a Philips Achieva 3T system (Best, ML) using a 32-channel cardiac coil as previously reported (Karolis et al., 2025). Each data set consists of 350 volumes (48 slices each) acquired using a single-shot echo planar imaging (EPI) (TR/TE = 2200/60) sequence, with slice matrix = 144 X 144, isotropic resolution = 2.2 mm, MB factor = 3, and SENSE factor = 1.4.

#### 2.3.2 dHCP neonatal studies

Neonatal studies were performed using a 3T Philips Achieva system (Edwards et al., 2022; Fitzgibbon et al., 2020). All infants were scanned without sedation in a scanner environment, including a dedicated transport system, positioning device, and a customized 32-channel receive coil with a custom-made acoustic hood. MRI-compatible ear putty and earmuffs provided additional acoustic noise attenuation, and infants were fed, swaddled, and positioned in a vacuum jacket prior to scanning to provide natural sleep. High temporal resolution multiband EPI (TE = 38 ms; TR = 392 ms; MB factor = 9x; 2.15 mm isotropic) specifically developed for neonates was acquired for 15 min.

### 2.4 Image processing

#### 2.4.1 Fetal connectivity preprocessing

fMRI data were preprocessed with the dHCP fetal functional pipeline (Karolis et al., 2025), including echo distortion correction, motion correction, independent component analysis (ICA) denoising, and registered to individual T2w native space. All further analyses were performed using BioImage Suite (Joshi et al., 2011) unless otherwise specified. Covariates of no interest regressed from the data included the following: 1) linear and quadratic drifts; 2) six motion parameters; 3) mean cerebral-spinal-fluid (CSF) signal; 4) mean white-matter signal; and 5) mean gray matter signal, as previously reported (Thomason et al., 2017). The data were temporally smoothed with a zero mean unit variance Gaussian filter (approximate cutoff frequency = 0.12 Hz). A gray-matter mask defined in template space was applied, and only gray matter voxels were used in further calculations.

#### 2.4.2 Neonatal connectivity preprocessing

fMRI data were preprocessed with the dHCP neonatal functional pipeline (Fitzgibbon et al., 2020), including echo distortion correction, motion correction, independent component analysis (ICA) denoising, and registered to individual T2w native space. All further analyses were performed using BioImage Suite (Joshi et al., 2011) unless otherwise specified. Covariates of no interest regressed from the data included the following: 1) linear and quadratic drifts; 2) mean cerebral-spinal-fluid signal; 3) mean white-matter signal; and 4) mean gray matter signal. For additional control of possible motion-related confounds, a 24-parameter motion model (including six rigid-body motion parameters, six temporal derivatives, and these terms squared) was regressed from the data. The data were temporally smoothed with a Gaussian filter (approximate cutoff frequency = 0.12 Hz).

#### 2.4.3 Seed pathway connectivity

*S*eed coordinates for this study were adult MNI coordinates adapted to the dHCP developmental templates (Chawarska et al., 2025; Lahnakoski et al., 2012). The time course for each seed region for each of the four proto pathways (Table 1, Fig. 1) was then computed as the average time course across all voxels in the reference region. The time courses were correlated between adjacent seed pairs in the pathways and transformed to z-values using Fisher’s transformation. As previously reported (Li et al., 2025), intra-pathway connectivity strength for each overall pathway was defined as the average of the individual connections within each, resulting in four intra-pathway connectivity strength values per individual. Inter-pathway connectivity strength between each pathway in the same hemisphere was defined as the connectivity between the pSTS and FFA (i.e., the key nodes in the social perception and ventral pathways) in each hemisphere, resulting in two values of inter-pathway connectivity strength per individual.

**Fig. 1.**
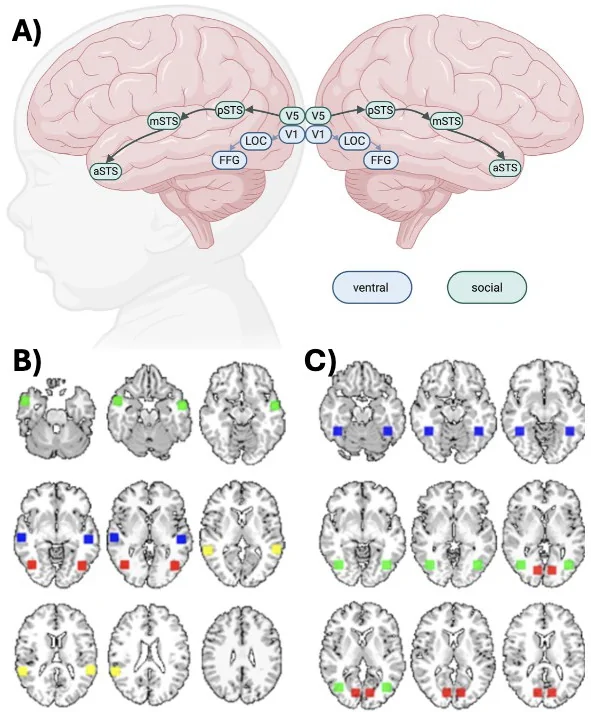
Schematic representation of the social perception and ventral face perception pathways in the brain. (A) The social perception pathway extends from the motion-sensitive medial temporal/visual 5 (V5) area through the posterior superior temporal sulcus (pSTS), medial STS (mSTS), and anterior STS (aSTS). The ventral object/face processing pathway extends from the primary visual cortex (V1) through the lateral occipital complex (LOC) to the fusiform gyrus (FFG) area. Panel A is reproduced from Chawarska et al. (2025). (B) The nodes for the social perception pathway: middle temporal area (red), posterior superior temporal sulcus (yellow), middle superior temporal sulcus (blue), and anterior superior temporal sulcus (green). (C) The nodes for the ventral pathway: V1 (red), lateral occipital complex (green), and fusiform gyrus (blue).

**Table 1.** MNI coordinates for social perception and ventral pathways.

Network	Nodes	Coordinates
Social	Right MTV5	42, -68, -6
	Left MTV5	-43, -70, -3
	Right pSTS	58, -44, 12
	Left pSTS	-58, -42, 12
	Right mSTS	60, -22, -2
	Left mSTS	-54, -25, -4
	Right aSTS	54, 1, -28
	Left aSTS	-56, -4, -16
Ventral	Right V1	12, -79, 6
	Left V1	-12, -79, 6
	Right LOC	40, -70, 0
	Left LOC	-40, -70, 0
	Right FFA	42, -50, -18
	Left FFA	-42, -50, -18

#### 2.4.4 Head motion

Since functional connectivity may be disturbed by head motion, we included several steps to ensure adequate control of motion confounds. Fetal data were censored for motion and data quality using DVARS values as reported by Karolis et al. (2025). In the neonatal sample, the mean frame-to-frame displacement was calculated for each run for every individual; runs with a mean frame-to-frame displacement of more than 0.2 mm were removed from further analysis. Iterative smoothing and regression of 24 motion parameters (six rigid-body parameters, six temporal derivatives of these parameters, and these 12 parameters squared) were used in the infant data. Finally, temporal signal-to-noise ratio (tSNR) data by PMA at scan are shown in Supplementary Figure S1; tSNR values were comparable across the study age range.

We anchored our analysis at 28, 34, 40, and 44 weeks PMA to accommodate the constraints of both our imaging capacities and the PMAs of the dHCP cohort and to highlight key neurodevelopmental milestones. The overall connectivity and short range and interhemispheric connections show sigmoid expansion peaking at the 26–29 week of gestation, with the inflection point at 27 weeks PMA (Jakab et al., 2014). This is also the point in gestation below which our registration algorithms cannot account for the lack of folding around the developing insula. The fetal brain triples in weight across the third trimester, and 34 weeks PMA represents a time of rapid strengthening of neural connections and the early formation of key neural networks in developing brain (Kostović et al., 2021; Thomason et al., 2014; Vasung et al., 2019). Term birth at 40 weeks marks the perinatal to neonatal transition and the exposure of the newborn brain to an entirely new range of experiences (Vasung et al., 2019). Finally, 44 weeks represents the close of imaging studies for the dHCP cohort and the end of the neonatal period.

### 2.5 Statistical analyses

The quantities of interest included iFC values and differences between iFC values, such as differences between homologous connectivities on the right versus the left side. To investigate longitudinal changes in these quantities as a function of PMA, we fit a Bayesian hierarchical generalized additive model (Wood, 2017) using the brms package in R (Burkner, 2017, 2018), which facilitates Bayesian inference using the probabilistic programming language Stan (Carpenter, 2017). The model used a Gaussian likelihood where both the mean connectivity and the residual standard deviation were estimated. The mean connectivity was modeled as a function of network and sex (fixed effects), with developmental trajectories taken to be penalized thin-plate regression splines with 15 basis functions applied to standardized PMA, allowing the developmental shape to vary by network, with random intercepts for each subject to account for repeated measurements of individuals. To allow for differences in signal variability across brain regions, the residual standard deviation was modeled as a function of network. Weakly informative priors were used for all parameters (default brms priors). Posterior probabilities were estimated using Hamiltonian Monte Carlo sampling, running 4 chains with 7000 iterations each (including 2000 warmup), resulting in 20,000 total post-warmup samples. For each quantity analyzed, the estimated parameters included mean values at the four target ages (28, 34, 40, and 44 weeks PMA), changes in the mean from one age to the next, and the total change over the study period. For each parameter, we report MCMC estimates for the posterior mean (denoted by “mean” or “M”) and 95% credible intervals (“CI”) with endpoints denoted by “lower” and “upper”. As an intuitive Bayesian analogue to traditional null hypothesis significance testing, we also quantify the certainty of our estimated effects by calculating the probability of a sign error (denoted by “pr”), defined as the posterior probability that the actual parameter has the opposite sign from its reported estimated mean. This metric aligns with the concept of “Type S” (Sign) errors advocated by Gelman and Carlin (2014), who argue that in continuous systems where true effects are rarely exactly zero, the probability of obtaining an effect in the wrong direction is more informative for scientific inference than testing a point-null hypothesis. This metric is also the mathematical complement of the well-known “probability of direction” (“pd”) of Makowski et al. (2019), who recommended reporting pd as their favored “index of effect existence,” with attractions including transparent interpretation and approximate relationship to the frequentist p-value (pr tends to approximate half of the two-sided p value). For example, a reported sign error probability of pr = 0.01 translates to pd = 1-0.01 = 0.99, that is, a 99% posterior probability that the parameter carries the estimated sign. Using Monte Carlo draws from the posterior distribution, pr is calculated as the proportion of these draws that fall on the opposite side of zero from the estimated posterior mean. As the probability of a sign error is one-sided, we report Bayesian statistical “significance” when pr is estimated to be less than 0.05/2 = 0.025.

Finally, to assess whether the observed developmental trajectories were robust to potential cross-sectional cohort effects, a sensitivity analysis applying the identical Bayesian hierarchical model was conducted exclusively on the “longitudinal subset” of participants who contributed both a fetal and a neonatal scan (see Supplemental Materials).

## 3 Results

### 3.1 Participants

Of 167 dHCP fetuses, 110 met study inclusion criteria; likewise, 477 of 800 Developing Human Connectome Project (dHCP) neonates met all inclusion criteria (Supplementary Table S1).

Sixty-five participants had a fetal scan without a neonatal scan, 432 participants had a neonatal scan without a fetal scan, and 45 participants had both fetal and neonatal scans. The number of fetal and neonatal subjects at each PMA are shown in Supplementary Table S2. Demographic characteristics for the fetuses and neonates are shown in Table 2. There were no significant differences in sex distribution (p = 0.748), PMA at birth (p = 0.818), or birth weight (p = 0.350) across the three groups. For the neonatal scans, the PMA at scan for the “both scans” group was larger than that of the “neonatal only” group (p < 0.001), likely reflecting differences in the logistics of scheduling. Because our regression framework models PMA continuously, such variations in scan timing are naturally incorporated in the estimated developmental trajectories.

**Table 2.** Sample demographics.

	Fetal only (n = 65)	Both scans (n = 45)	Neonatal only (n = 432)	p value*
Males	35 (53.8%)	23 (51.1%)	244 (56.5%)	0.748
Birth weight (kg)	3.38 (0.45)	3.50 (0.42)	3.44 (0.45)	0.350
PMA at birth (weeks)	40.16 (1.21)	40.19 (1.22)	40.09 (1.14)	0.818
PMA at fetal scan (weeks)	31.44 (2.53)	31.66 (2.95)	-	0.694
PMA at neonatal scan (weeks)	-	42.41 (1.43)	41.31 (1.57)	<0.001

* p-values from Chi-square (sex), ANOVA (birth weight, PMA at birth), or two-sample t-tests (scan ages).

### 3.2 Ethics

The Developing Human Connectome Project (dHCP) was approved by the UK Health Research Authority Research Ethics Committee (REC reference 14/LO/1169), and written informed consent was obtained from the parents or legal guardians of all participants for imaging and open-data release of anonymized data. The present study involved secondary analysis of de-identified data obtained from the dHCP public repository.

### 3.3 Functional imaging analysis

#### 3.3.1 Sex effects

We found no significant sex effects within any of the six networks, as shown in Supplementary Table S3. Our estimates for network connectivity in all results shown below are unweighted averages of the estimates for females and males.

#### 3.3.2 Intra-pathway connectivity

The intra-pathway connectivity was computed as the average of correlations between adjacent seed pairs within each pathway (see Section 2). As shown in Tables 3 and 4, comparisons of the pathway iFC to zero showed that during the prenatal and the neonatal periods, both pathways in both hemispheres have iFC significantly greater than 0 (pr < 0.001 for all).

**Table 3.** Proto social perception pathway iFC.

Left proto social pathway				
Estimated quantity	Mean	Lower	Upper	pr (sign error)
PMA = 28	0.1931	0.1387	0.2509	0.00000
PMA = 34	0.1874	0.1445	0.2260	0.00000
PMA = 40	0.2542	0.2335	0.2736	0.00000
PMA = 44	0.3148	0.2830	0.3449	0.00000
(PMA = 34)-(PMA = 28)	-0.0056	-0.0835	0.0621	0.45590
(PMA = 40)-(PMA = 34)	0.0667	0.0267	0.1111	0.00240
(PMA = 44)-(PMA = 40)	0.0606	0.0237	0.0991	0.00120
(PMA = 44)-(PMA = 28)	0.1217	0.0554	0.1833	0.00045
Right proto social pathway				
Estimated quantity	Mean	Lower	Upper	pr (sign error)
PMA = 28	0.1486	0.0885	0.2084	0.00005
PMA = 34	0.1208	0.0614	0.1720	0.00020
PMA = 40	0.2741	0.2522	0.2968	0.00000
PMA = 44	0.3745	0.3410	0.4079	0.00000
(PMA = 34)-(PMA = 28)	-0.0278	-0.1145	0.0557	0.26700
(PMA = 40)-(PMA = 34)	0.1533	0.0991	0.2201	0.00000
(PMA = 44)-(PMA = 40)	0.1004	0.0586	0.1418	0.00005
(PMA = 44)-(PMA = 28)	0.2259	0.1568	0.2941	0.00000

The upper four rows display estimates of the mean, lower, and upper endpoints of 95% probability credible intervals, and the probability of a sign error for the within-pathway connectivity values at postmenstrual ages of 28, 34, 40, and 44 weeks. The lower four rows show the same estimates for the increases in intra-pathway connectivity from 28 to 34 weeks, from 34 to 40 weeks, from 40 to 44 weeks, and from 28 to 44 weeks postmenstrual age.

**Table 4.** Proto ventral pathway iFC.

Left proto ventral pathway				
Estimated quantity	Mean	Lower	Upper	pr (sign error)
PMA = 28	0.0794	0.0320	0.1230	0.00135
PMA = 34	0.1153	0.0860	0.1560	0.00000
PMA = 40	0.1317	0.1146	0.1478	0.00000
PMA = 44	0.1486	0.1229	0.1730	0.00000
(PMA = 34)-(PMA = 28)	0.0359	-0.0116	0.1034	0.06605
(PMA = 40)-(PMA = 34)	0.0163	-0.0280	0.0449	0.15565
(PMA = 44)-(PMA = 40)	0.0169	-0.0130	0.0471	0.10980
(PMA = 44)-(PMA = 28)	0.0692	0.0183	0.1215	0.00395
Right proto ventral pathway				
Estimated quantity	Mean	Lower	Upper	pr (sign error)
PMA = 28	0.1965	0.1200	0.2716	0.00000
PMA = 34	0.1202	0.0662	0.1797	0.00005
PMA = 40	0.1935	0.1700	0.2177	0.00000
PMA = 44	0.2193	0.1822	0.2549	0.00000
(PMA = 34)-(PMA = 28)	-0.0763	-0.1679	0.0177	0.05295
(PMA = 40)-(PMA = 34)	0.0733	0.0097	0.1316	0.01385
(PMA = 44)-(PMA = 40)	0.0258	-0.0197	0.0702	0.12900
(PMA = 44)-(PMA = 28)	0.0227	-0.0635	0.1083	0.30100

The upper four rows display estimates of the mean, lower and upper endpoints of 95% probability credible intervals, and the probability of a sign error for the within-pathway connectivity values at postmenstrual ages of 28, 34, 40, and 44 weeks. The lower four rows show the same estimates for the increases in intra-pathway connectivity from 28 to 34 weeks, from 34 to 40 weeks, from 40 to 44 weeks, and from 28 to 44 weeks postmenstrual age.

Longitudinal contrasts comparing connectivity strengths over the four time points (Tables 3 and 4; Fig. 2, Supplementary Figs. S2 and S3) revealed significant increases in connectivity across the pre- to post-natal transition (PMA44–PMA28) in the left pSP: M = 0.122, the right pSP: M = 0.226, and the left pVP: M = 0.069 (pr < 0.001, pr < 0.001and pr = 0.004, respectively) but not for the right pVP: M = 0.023 (pr = 0.301). As shown in Table 3, Table 4, and Figure 2, the changes occurred at different schedules in different pathways. In the left pSP, there were signiﬁcant increases from PMA34 to PMA40 and PMA40 to PMA44 (M = 0.067 and M = 0.061; pr = 0.002 and pr = 0.001, respectively). Likewise, there were also signiﬁcant increases from PMA34 to PMA40 and from PMA40 to PMA44 in the right pSP (M = 0.153 and M = 0.10; pr < 0.001 for both). There was no signiﬁcant increase from PMA28 to PMA34 in either pSP (left M = -0.006; right M = -0.028; pr = 0.456 and pr = 0.267, respectively).

**Fig. 2.**
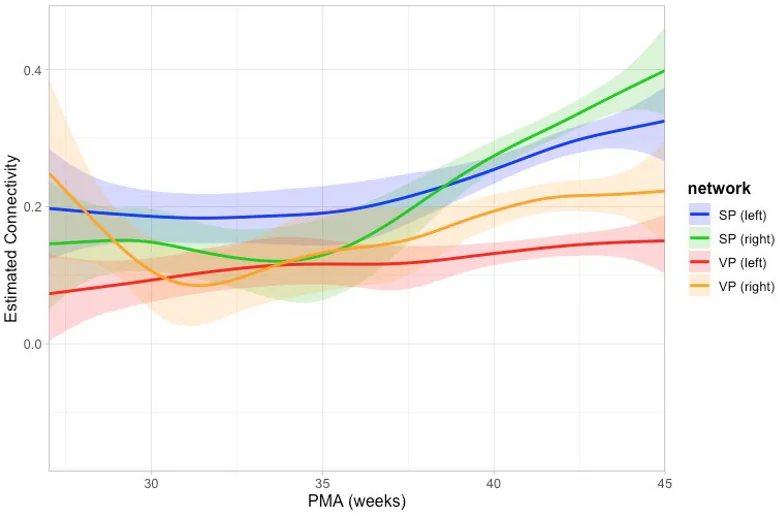
Developmental trajectories of the social perception pathways (pSP) and ventral pathways (pVP) with 95% credible intervals.

There were no significant differences from PMA28 to PMA34, PMA34 to PMA40, and PMA40 to PMA44 in the left pVP (M = 0.036, M = 0.016, and M = 0.017; pr = 0.066, pr = 0156, pr = 0.110, respectively). For the right pVP there was no significant change from PMA28 to PMA34 (M = -0.076; pr = 0.052), a significant increase between PMA34 and PMA40 (M = 0.073; pr = 0.014), and no significant change between PMA40 and PMA44 (M = 0.026; pr = 0.129).

#### 3.3.3 Within-pathway hemispheric differences

We next compared the developmental trajectories of the pSP and pVP in the left and right hemispheres (Supplementary Table S4). In the pSP, iFC became higher in the right hemisphere than that in the left hemisphere during the late third trimester. While connectivity was significantly lower on the right side at 34 weeks (M = -0.067, pr = 0.022), an increase in right-sided iFC between 34 and 40 weeks (M = 0.087, pr = 0.006) reversed this asymmetry, resulting in significantly higher right iFC by 44 weeks (M = 0.060, pr = 0.003). The right-dominance of the pSP increased significantly between 28 and 44 weeks (M = 0.104, pr = 0.011). In contrast, the pVP development showed no reversal of asymmetry, with iFC higher in the right versus left pVP across the study period (significant at 28, 40, and 44 weeks, all pr ≤ 0.003). There was no evidence for changes in the right hemisphere dominance in the pVP between 28 and 44 weeks (M = -0.046, pr = 0.175). This suggests that the higher iFC values in the right pVP are established early and maintained consistently, whereas the pSP undergoes a reversal, showing higher iFC in the left hemisphere early in the third trimester and higher iFC values in the right hemisphere during the fetal to neonatal transition.

#### 3.3.4 Between-pathway comparisons

The analysis shown in Table 5 compared directly the iFC values between the pSP and pVP in both hemispheres. In the left hemisphere, the iFC was higher in the pSP than the pVP at all 4 time points (PMA28, PMA34, PMA40, PMA44: M = 0.114, 0.072, 0.122, 0.166; pr = < 0.001, 0.006, < 0.001, < 0.001, respectively). In contrast, in the right hemisphere there was no difference in the pSP versus pVP at PMA28 and PMA34 (M = -0.048, 0.001; pr = 0.153, 0.480, respectively), but IFC was significantly higher in the pSP than in the pVP at both PMA40 and PMA44 (M = 0.081, 0.155; pr < 0.001 for both).

**Table 5.** Differences in the pathway intrinsic functional connectivity between the two hemispheres in the proto social pathway (pSP) and proto ventral pathway (pVP).

pSP left - pVP left				
Estimated quantity	Mean	Lower	Upper	pr (sign error)
PMA = 28	0.1136	0.0464	0.1843	0.00030
PMA = 34	0.0721	0.0178	0.1204	0.00605
PMA = 40	0.1225	0.0979	0.1470	0.00000
PMA = 44	0.1662	0.1284	0.2034	0.00000
(PMA = 34)-(PMA = 28)	-0.0415	-0.1356	0.0413	0.18410
(PMA = 40)-(PMA = 34)	0.0504	0.0006	0.1079	0.02380
(PMA = 44)-(PMA = 40)	0.0436	-0.0013	0.0891	0.02860
(PMA = 44)-(PMA = 28)	0.0526	-0.0279	0.1298	0.09930
pSP right - pVP right				
Estimated quantity	Mean	Lower	Upper	pr (sign error)
PMA = 28	-0.0479	-0.1398	0.0440	0.15285
PMA = 34	0.0006	-0.0795	0.0727	0.48030
PMA = 40	0.0806	0.0495	0.1116	0.00000
PMA = 44	0.1552	0.1088	0.2019	0.00000
(PMA = 34)-(PMA = 28)	0.0485	-0.0718	0.1665	0.21400
(PMA = 40)-(PMA = 34)	0.0800	0.0011	0.1679	0.02345
(PMA = 44)-(PMA = 40)	0.0746	0.0173	0.1324	0.00570
(PMA = 44)-(PMA = 28)	0.2031	0.0994	0.3079	0.00005

The upper four rows display estimates of the mean, lower and upper endpoints of 95% probability credible intervals, and the probability of a sign error for the pSP – pVP pathway differences in connectivity at postmenstrual ages of 28, 34, 40, and 44 weeks. The lower four rows show the same estimates for the differences in pSP - pVP pathway connectivity from 28 to 34 weeks, from 34 to 40 weeks, from 40 to 44 weeks, and from 28 to 44 weeks postmenstrual age.

### 3.4 Secondary analyses

#### 3.4.1 FFA and pSTS connectivity

Next, we examined iFC between the two key nodes of the pVP and pSP, the FFA and the pSTS (Table 6, Fig. 3 and Supplementary Table S5). While connectivity was not significantly different from 0 in both hemispheres at 28 and 34 weeks, the trajectories diverged significantly thereafter. The inter-connectivity in the right hemisphere became positive at 40 weeks (M = 0.028, pr = 0.015) and 44 weeks (M = 0.097, pr < 0.001) and in the left hemisphere it became negative by 44 weeks (M = -0.065, pr < 0.002).

**Fig. 3.**
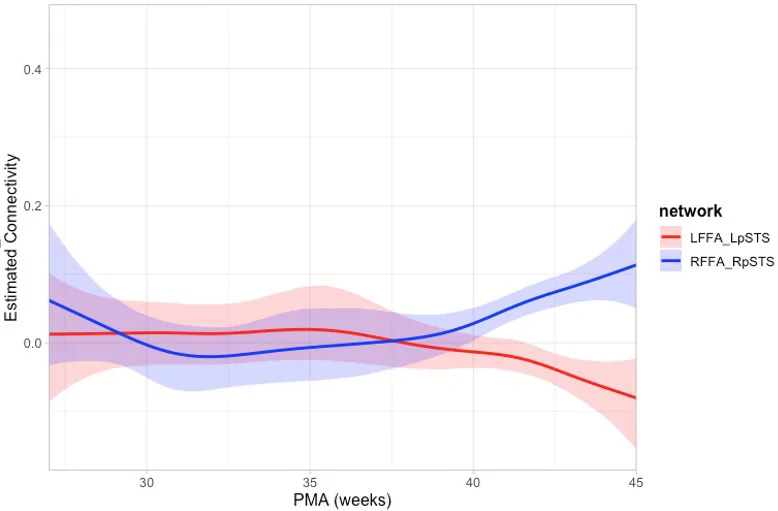
Developmental trajectories of FFA and the pSTS connectivity with 95% credible intervals.

**Table 6.** FFA to pSTS intrinsic functional connectivity.

Left FFA - left pSTS				
Estimated quantity	Mean	Lower	Upper	pr (sign error)
PMA = 28	0.0131	-0.0540	0.0765	0.33515
PMA = 34	0.0182	-0.0259	0.0725	0.21810
PMA = 40	-0.0133	-0.0369	0.0115	0.13885
PMA = 44	-0.0647	-0.1075	-0.0277	0.00015
(PMA = 34)-(PMA = 28)	0.0052	-0.0684	0.0952	0.48640
(PMA = 40)-(PMA = 34)	-0.0315	-0.0884	0.0167	0.08705
(PMA = 44)-(PMA = 40)	-0.0515	-0.1044	-0.0077	0.00835
(PMA = 44)-(PMA = 28)	-0.0778	-0.1524	-0.0057	0.01720
Right FFA – right pSTS				
Estimated quantity	Mean	Lower	Upper	pr (sign error)
PMA = 28	0.0398	-0.0268	0.1102	0.12310
PMA = 34	-0.0114	-0.0581	0.0418	0.30380
PMA = 40	0.0280	0.0030	0.0508	0.01510
PMA = 44	0.0969	0.0620	0.1329	0.00000
(PMA = 34)-(PMA = 28)	-0.0512	-0.1374	0.0293	0.11165
(PMA = 40)-(PMA = 34)	0.0394	-0.0210	0.0895	0.07675
(PMA = 44)-(PMA = 40)	0.0689	0.0257	0.1137	0.00080
(PMA = 44)-(PMA = 28)	0.0571	-0.0223	0.1295	0.07475

The upper four rows display estimates of the mean, lower and upper endpoints of 95% probability credible intervals, and the probability of a sign error for the within-pathway connectivity values at postmenstrual ages of 28, 34, 40, and 44 weeks. The lower four rows show the same estimates for the increases in intra-pathway connectivity from 28 to 34 weeks, from 34 to 40 weeks, from 40 to 44 weeks, and from 28 to 44 weeks postmenstrual age.

## 4 Discussion

This study demonstrates for the first time the robust presence of proto social perception and ventral face processing pathways across the third trimester of gestation and perinatal transition and their right-ward lateralization. Despite these similarities, the pathways differed in their developmental dynamics with the proto social pathway demonstrating a more rapid rise in connectivity reaching the highest level of connectivity by 44 weeks compared to all other pathways. The study also demonstrates divergence in connectivity patterns between the key nodes in both networks, pSTS and FFA, with strengthening of the connectivity in the right hemisphere and weakening in the left hemisphere. Together, these findings demonstrate that neural architecture supporting face and multimodal social signal processing observed typically in older children, is present in an elementary form in the third trimester and undergoes rapid development across the pre- to postnatal transition. The strengthening of intrinsic connectivity, emergence of right-hemisphere dominance and early differentiation of putative social perception and face-processing circuitry indicate that key features of the social brain begin to assemble before birth, presumably providing a developmental scaffold for later social development.

Unlike other higher order cortical networks such as the salience or language networks that demonstrate little evidence for neural connectivity across the early and mid-third trimester (Scheinost et al., 2021, 2024), the proto social perception and ventral pathways demonstrate positive within-pathway connectivity already at 28 weeks PMA. The functional connectivity increases subsequently in the left and right pSP and in the left pVP, presumably secondary to age-related changes in strength and length of the connections and the network reorganization that may accompany the perinatal transition (Desrosiers et al., 2024; Fair et al., 2009; Ji et al., 2024; Zhao et al., 2019). Both proto pathways are robustly right lateralized during postnatal period, which may reflect a general lateralization trend for most networks in term neonates (Bisiacchi & Cainelli, 2022). Consistent with other fetal and neonatal resting-state functional studies (Bisiacchi & Cainelli, 2022), we found no evidence for sex dimorphism in developmental dynamics or lateralization patterns of the proto pathways.

While the two share key similarities, the putative pathways involved in processing of faces and social engagement signals diverge when it comes to their differential rates of growth and lateralization patterns. While connectivity was higher in the left pSP at 34 weeks, a relative surge in right-sided iFC between 34 and 40 weeks reversed this asymmetry, resulting in significantly higher iFC by 44 weeks. Differential structural maturation of the right proto STS region was observed early in the fetal stage of development, as it appears up to 2 weeks earlier than the left STS (de Vareilles et al., 2023; Habas et al., 2012; Kasprian et al., 2011; Yun et al., 2022), and thus may mature faster than its homologous counterpart. By 44 weeks PMA, iFC in the right pSP exceeds connectivity observed in remaining pathways owing to a rapid increase during the pre- to postnatal transition. In contrast, the pVP connectivity was significantly greater on the right side than the left throughout most of the study period, with no significant difference between the hemispheres in the amount of growth across the 28- to 44-week period. The right-ward asymmetries in functional connectivity of the temporal lobe observed here mirror those reported in fetal cortical folding and volumetric studies in addition to both microstructural and functional analyses (Bisiacchi & Cainelli, 2022; Habas et al., 2012; Kasprian et al., 2011).

We also examined iFC between the FFA and pSTS regions. Task-based fMRI evidence suggests that both the STS and FFA respond selectively to faces in 2- to 9-month-old infants and that this response is present even in the youngest infants, presumably enabling infants to actively decode their environment and secure their needs within an inherently social world (Kosakowski et al., 2024; Saxe & Kosakowski, 2026). In the adult brain, the right pSTS and FFA exhibit robust resting-state functional connectivity, revealing that face processing emerges from the synergy of distributed, yet highly specialized, neural circuits (Turk-Browne et al., 2010). Here, we report that this synergy may originate very early in development. Specifically, iFC between the right pSTS and pFFA has reached positive values at 40 weeks and strengthened further at 44 weeks PMA. In contrast, connectivity between the homologous regions in the left hemisphere became increasingly negative by 44 weeks PMA. Together, these findings suggest emerging hemispheric differentiation in inter-pathway coupling, with the right hemisphere showing increasing integration between regions implicated in social perception and face processing, while the homologous regions in left hemisphere become increasingly decoupled. This early divergence may represent an initial step toward hemispheric specialization of social perceptual circuitry, although the functional significance of these emerging asymmetries remains to be determined.

The evidence for potential significance of the rapid development of the right proto pathways during the neonatal period is only beginning to emerge. A recent study examining social perception and ventral pathways in a sample of neonates followed prospectively between 1 and 4 months, demonstrated robust links between the iFC in the right social pathway in neonates with attention to dynamic multimodal faces at 4 months, which, in turn, predicted more advanced social behaviors at 12-18 months (Chawarska et al., 2025). This effect was not present when connectivity in the left social or ventral pathways was examined. The developmental significance of these findings is further underscored by a recent report suggesting links between hypoconnectivity within the right social pathway and symptoms of autism in school-age children (Li et al., 2024). Here, we demonstrate that the putative precursors of the pathways involved in processing faces and voices begin to form long before birth, providing motivation for future studies examining individual differences in the pathway dynamics and later outcomes relevant to well-being and adaptation.

Specifically, numerous studies associate fetal exposures with the neonatal connectome (De Asis-Cruz et al., 2021; Spann et al., 2018; Sylvester et al., 2018; Wu et al., 2024), but the predictive value of the fetal connectome for later outcomes including the neonatal period is just beginning to be explored. Recent data suggest iFC between the fetal supplementary motor area and visual cortices at 25 to 39 weeks PMA is associated with motor function at 7 months postnatal age (Ji et al., 2023), weaker functional coupling between the subcortical limbic network and medial prefrontal network in fetuses has been associated with greater child aggression at age 3 years (Hendrix et al., 2023), and fetal networks may foreshadow the onset of neurodevelopmental conditions (De Asis-Cruz & Limperopoulos, 2023). Future studies will be necessary to determine if there are links between individual differences in development of the proto social perception pathways in the third trimester and later outcomes.

Key strengths of the work are the stringent inclusion and exclusion criteria, PMA-appropriate MRI templates, the robust sample size, and the combined fetal-neonatal developmental window. However, the latter can also be seen as a potential limitation positing interpretive challenges. The fetal and neonatal data were acquired with different scan parameters and were processed with different pipelines tailored to each population, raising concerns about differences in denoising, motion structure, temporal resolution, or residual noise characteristics across the two modalities (Karolis et al., 2025; Thomason, 2020; Thomason et al., 2017). To ensure that our findings reflect true within-subject maturation rather than cross-sectional cohort differences, we conducted a sensitivity analysis on the longitudinal subset of 45 participants who contributed both a fetal and a neonatal scan, which successfully replicated the primary developmental trajectories (see Supplementary Materials, Tables S6 and S7). The risk of a global pipeline artifact (e.g., an artificial uniform inflation of neonatal connectivity scores relative to fetal scans) is further mitigated by the pathway-specific and divergent trajectories observed in both our full sample and the within-subject longitudinal sample, with the left pSP, right pSP, and left pVP increasing strongly across the perinatal transition while the right pVP did not increase. These considerations support a biological interpretation of differential network maturation.

Our study suggests that the proto networks mapping structurally onto those brain areas implicated in later social perceptual functioning and face processing are already detectable during the late prenatal period and continue to mature across the transition to postnatal life. The strengthening of intrinsic connectivity within the proto social perception pathway, emergence of right-hemisphere dominance pattern observed in older individuals, and interconnectivity with the proto face processing region indicate that putative neural systems that will later support social information processing begin to organize before birth. These findings advance our understanding of the developmental origins of social brain networks and motivate future studies examining whether variability in the development of these proto networks during the third trimester relates to neonatal functioning and whether their developmental dynamics forecast later social adaptation and well-being in early childhood.

## Supplementary Material

Supplementary Material

## Data Availability

The image analysis software (BioImage Suite) can be found at https://medicine.yale.edu/bioimaging/suite/ and https://bioimagesuiteweb.github.io/webapp/index.html
