# Supplementary material for: Development of the social perception pathway across the third trimester of gestation and perinatal transition
Source: Imaging Neurosci (Camb). 2026 Oct 15;4:IMAG.a.1394. doi: 10.1162/IMAG.a.1394 (PMC13673308; doi:10.1162/IMAG.a.1394)
Supplement: Supplementary file 1 [file IMAG.a.1394_supp.pdf]

SUPPLEMENT

Table S1. Sample Reduction: Exclusion Criteria

	Fetuses (n = 167)		Term Neonates (n = 800)	
**Exclusion Criteria**	Removed (count)	Remaining (count)	Removed (count)	Remaining (count)
**Non-singleton**	0	167	122	678
**Missing PMA birth**	3	164	0	678
**Birth PMA < 37 weeks**	4	160	181	497
**Missing BW**	49	111	0	497
**BW < 2.5 kg**	1	110	15	482
**Structural changes**	0	110	5	477
**Final sample**		110		477

Table S2. Sample size for fetuses and neonates for each PMA bin.

PMA (week)	Fetuses (count)	Term Neonates (count)
**27**	8	0
**28**	16	0
**29**	16	0
**30**	8	0
**31**	12	0
**32**	18	0
**33**	11	0
**34**	6	0
**35**	6	0
**36**	6	0
**37**	2	2
**38**	1	27
**39**	0	65
**40**	0	99
**41**	0	104
**42**	0	81
**43**	0	71
**44**	0	28
**Total**	110	477

Table S3. Estimated sex effects

network	mean	lower	upper	pr(sign error)
**VP left**	-0.0183	-0.0423	0.0059	0.06985
**VP right**	-0.0064	-0.0344	0.0213	0.32805
**LFFA_LpSTS**	0.0064	-0.0254	0.0382	0.34590
**RFFA_RpSTS**	-0.0088	-0.0376	0.0204	0.27045
**SP left**	0.0170	-0.0095	0.0431	0.10225
**SP right**	-0.0094	-0.0366	0.0177	0.24675

Legend: The "mean" column gives posterior mean differences in the order male - female.

Table S4. Comparison of Right and Left Proto Social and Ventral Pathways

Right pSP – Left pSP

estimated quantity	mean	lower	upper	pr(sign error)
**PMA=28**	-0.0445	-0.1243	0.0335	0.13370
**PMA=34**	-0.0667	-0.1352	-0.0019	0.02150
**PMA=40**	0.0199	-0.0080	0.0485	0.08000
**PMA=44**	0.0597	0.0163	0.1035	0.00310
**(PMA=34)-(PMA=28)**	-0.0222	-0.1294	0.0859	0.34445
**(PMA=40)-(PMA=34)**	0.0865	0.0181	0.1615	0.00600
**(PMA=44)-(PMA=40)**	0.0398	-0.0141	0.0927	0.07360
**(PMA=44)-(PMA=28)**	0.1042	0.0148	0.1949	0.01115

Right pVP – Left pVP

estimated quantity	mean	lower	upper	pr(sign error)
**PMA=28**	0.1171	0.0322	0.2012	0.00320
**PMA=34**	0.0049	-0.0584	0.0715	0.44280
**PMA=40**	0.0618	0.0347	0.0896	0.00000
**PMA=44**	0.0707	0.0285	0.1122	0.00045
**(PMA=34)-(PMA=28)**	-0.1122	-0.2170	-0.0076	0.01775
**(PMA=40)-(PMA=34)**	0.0570	-0.0137	0.1258	0.05200
**(PMA=44)-(PMA=40)**	0.0088	-0.0429	0.0588	0.36680
**(PMA=44)-(PMA=28)**	-0.0464	-0.1429	0.0493	0.17460

Legend: The upper four rows display estimates of the mean, lower and upper endpoints of 95% probability credible intervals, and the probability of a sign error for the Right – Left pathway connectivity values at postmenstrual ages of 28, 34, 40, and 44 weeks. The lower four rows show the same estimates for the increases in intra-pathway connectivity from 28 to 34 weeks, from 34 to 40 weeks, from 40 to 44 weeks and from 28 to 44 weeks postmenstrual age.

Table S5. Comparison of Right and Left FFA-pSTS connectivity

Right FFA–Right pSTS – Left FFA-Left pSTS

estimated quantity	mean	lower	upper	pr(sign error)
**PMA=28**	0.0267	-0.0622	0.1201	0.28425
**PMA=34**	-0.0297	-0.0991	0.0379	0.18465
**PMA=40**	0.0413	0.0085	0.0730	0.00765
**PMA=44**	0.1616	0.1115	0.2145	0.00000
**(PMA=34)-(PMA=28)**	-0.0564	-0.1748	0.0526	0.16100
**(PMA=40)-(PMA=34)**	0.0710	-0.0026	0.1439	0.02810
**(PMA=44)-(PMA=40)**	0.1204	0.0586	0.1861	0.00000
**(PMA=44)-(PMA=28)**	0.1349	0.0319	0.2345	0.00555

Legend: The upper four rows display estimates of the mean, lower and upper endpoints of 95% probability credible intervals, and the probability of a sign error for the within-pathway connectivity values at postmenstrual ages of 28, 34, 40, and 44 weeks. The lower four rows show the same estimates for the increases in intra-pathway connectivity from 28 to 34 weeks, from 34 to 40 weeks, from 40 to 44 weeks and from 28 to 44 weeks postmenstrual age.

Postmenstrual ages of the data presentation in the tables

Data are presented at 28, 34, 40 and 44 weeks PMA to respect the constraints of both our imaging capacities and the PMAs of the dHCP cohort. Twenty-seven and one-half weeks post menstrual age (PMA) is the age below which our registration algorithms cannot account for the lack of folding around the developing insula. Forty-four weeks is the age of the oldest infant in the dHCP cohort, and 40 weeks PMA marks the perinatal to neonatal transition. Thirty-four weeks PMA is mid-point between 28 and 40 weeks.

Longitudinal subset analysis

Forty-five participants contributed both a fetal scan and a neonatal scan. The within-subject nature of this longitudinal subset inherently controls for potential cross-sectional cohort effects. We therefore used this analysis to check that our main results would also be reflected (although presumably with less power) in this subset.

We found that the results in the longitudinal subgroup were similar to the results from the full data set, with our key findings replicated. The most fundamental findings for the Social Perception (pSP) pathway and the Ventral Pathway (pVP) from the full data included robust pre- to postnatal increases in the left pSP, right pSP, and the left pVP, contrasted by a lack of increase in the right pVP. In the submitted article, these results are shown in Table 4 and Table 5; see in particular the "(PMA=44)-(PMA=28)" rows. The two tables shown below, Table S6 and Table S7, are the longitudinal-subset counterparts of Table 3 and Table 4, displaying the results of fitting the same model to the longitudinal subset. These within-subject results show the same pattern as the full data, with the "(PMA=44)-(PMA=28)" fetal-to-neonatal increase being positive in the left pSP (mean = .2197, pr < .001), right pSP (mean = .2559, pr < .001), and left pVP (mean = .1256, pr = .007), but no increase in the right pVP (mean = −.0405, pr = .312).

If the observed developmental changes were driven by cohort differences or systematic differences in acquisition parameters, temporal resolution, denoising choices, motion structures, or residual noise characteristics across the two modalities, we would expect to see a consistent global effect, such as an artificial relative inflation of connectivity scores at the neonatal time point or changes that fail to replicate within individuals. Such global effects are not consistent with the pathway-specific increases (or lack thereof) observed within these

individuals. Across the perinatal transition, finding some pathways increasing strongly while others remain flat provides evidence that these results reflect within-subject differential biological development rather than cohort effects, preprocessing, or collection artifacts.

Table S6. Results for longitudinal subset: Proto Social Pathway

Left Proto Social Pathway

estimated quantity	mean	lower	upper	pr(sign error)
**PMA=28**	0.1141	0.0420	0.1913	0.00135
**PMA=34**	0.1815	0.1227	0.2356	0.00000
**PMA=40**	0.2740	0.2220	0.3287	0.00000
**PMA=44**	0.3338	0.2757	0.3924	0.00000
**(PMA=34)-(PMA=28)**	0.0674	-0.0332	0.1507	0.07915
**(PMA=40)-(PMA=34)**	0.0925	0.0319	0.1629	0.00450
**(PMA=44)-(PMA=40)**	0.0598	-0.0171	0.1317	0.04770
**(PMA=44)-(PMA=28)**	0.2197	0.1220	0.3122	0.00000

Right Proto Social Pathway

estimated quantity	mean	lower	upper	pr(sign error)
**PMA=28**	0.1479	0.0586	0.2347	0.00065
**PMA=34**	0.1032	0.0206	0.1800	0.00830
**PMA=40**	0.2623	0.1874	0.3363	0.00000
**PMA=44**	0.4038	0.3327	0.4736	0.00000
**(PMA=34)-(PMA=28)**	-0.0447	-0.1647	0.0741	0.23125
**(PMA=40)-(PMA=34)**	0.1591	0.0620	0.2682	0.00115
**(PMA=44)-(PMA=40)**	0.1415	0.0349	0.2451	0.00555
**(PMA=44)-(PMA=28)**	0.2559	0.1464	0.3647	0.00005

Table S7. Results for longitudinal subset: Proto Ventral Pathway

Left Proto Ventral Pathway

estimated quantity	mean	lower	upper	pr(sign error)
**PMA=28**	0.0500	-0.0309	0.1280	0.11140
**PMA=34**	0.1351	0.0656	0.2234	0.00005
**PMA=40**	0.0995	0.0240	0.1646	0.00575
**PMA=44**	0.1756	0.1119	0.2450	0.00000
**(PMA=34)-(PMA=28)**	0.0851	-0.0199	0.2083	0.06075
**(PMA=40)-(PMA=34)**	-0.0357	-0.1575	0.0533	0.29775
**(PMA=44)-(PMA=40)**	0.0762	-0.0137	0.1874	0.05270
**(PMA=44)-(PMA=28)**	0.1256	0.0233	0.2356	0.00735

Right Proto Ventral Pathway

estimated quantity	mean	lower	upper	pr(sign error)
**PMA=28**	0.2088	0.0770	0.3369	0.00070
**PMA=34**	0.1189	0.0142	0.2375	0.01285
**PMA=40**	0.2430	0.1432	0.3490	0.00000
**PMA=44**	0.1683	0.0738	0.2604	0.00050
**(PMA=34)-(PMA=28)**	-0.0899	-0.2500	0.0692	0.13905
**(PMA=40)-(PMA=34)**	0.1242	-0.0243	0.2672	0.04745
**(PMA=44)-(PMA=40)**	-0.0748	-0.2232	0.0686	0.16375
**(PMA=44)-(PMA=28)**	-0.0405	-0.2054	0.1323	0.31190

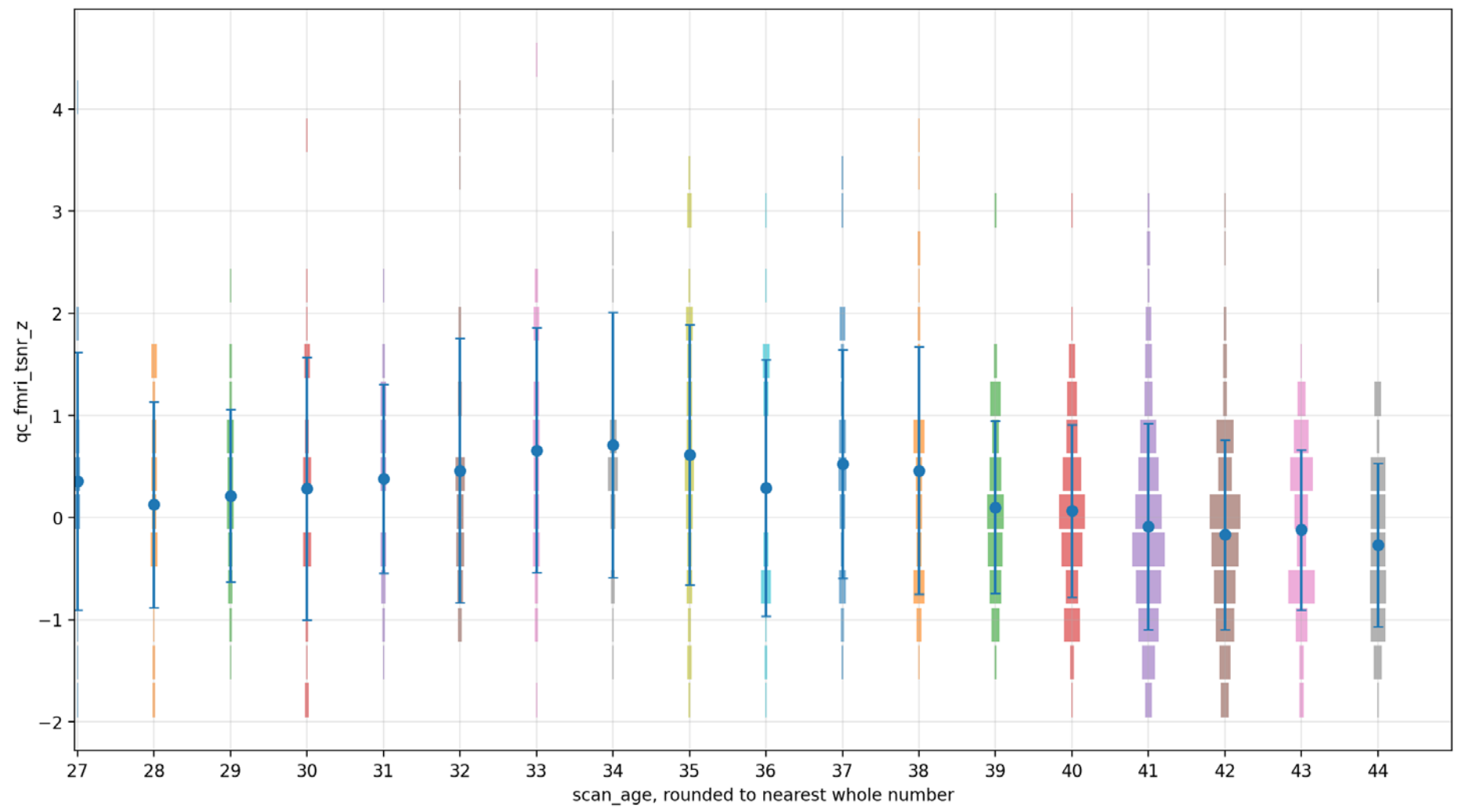

Figure S1. Temporal signal-to-noise ratios (tSNR) for fetuses and neonates for each PMA bin

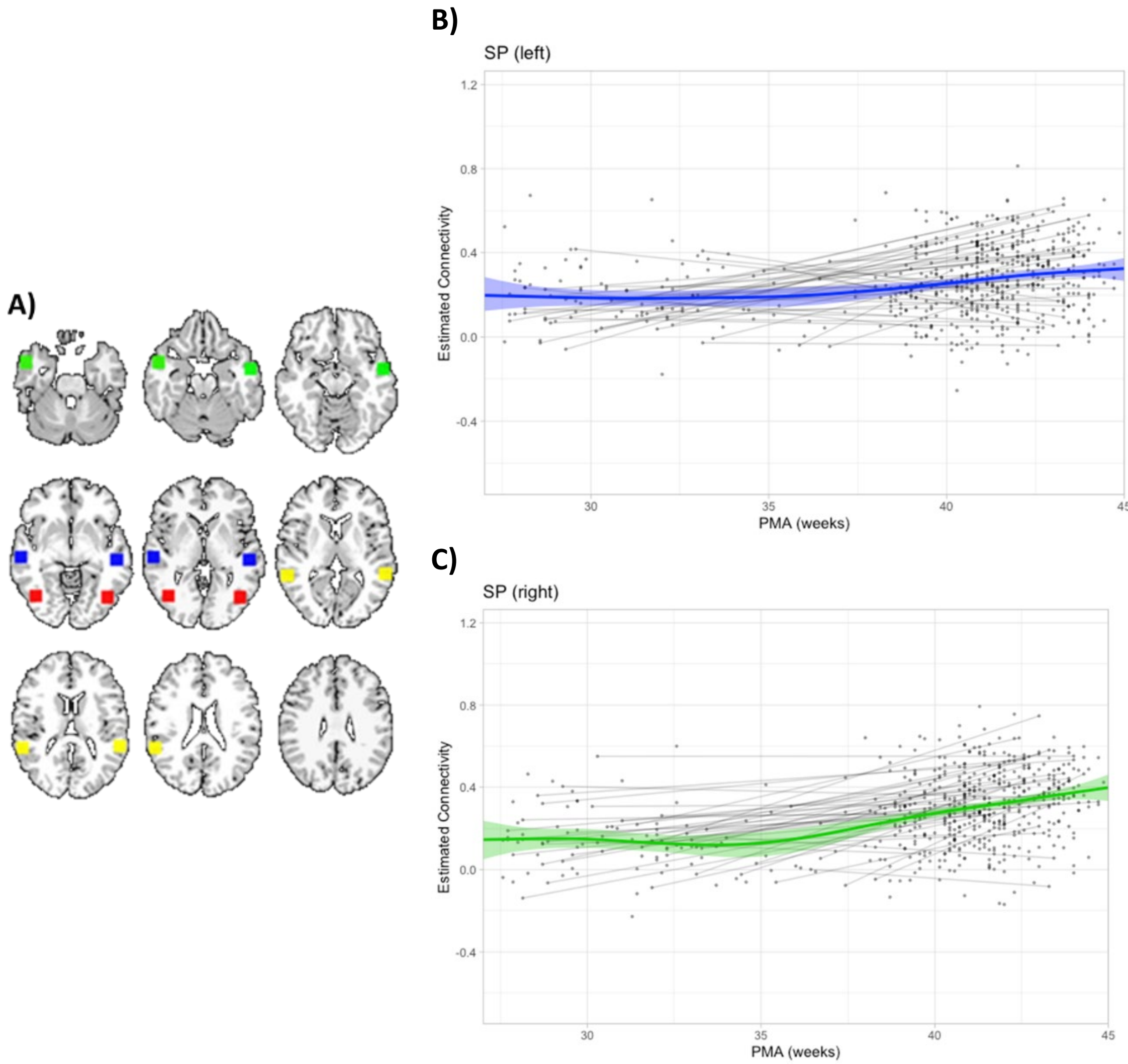

Figure S2. Longitudinal development of the social perception pathway across the third trimester of gestation and perinatal transition including all data points. A) The nodes for the social perception pathway: middle temporal area (red), posterior superior temporal sulcus (yellow), middle superior temporal sulcus (blue), and anterior superior temporal sulcus (green). B) Trajectory of the intra-pathway connectivity of the social perception pathway for the left hemisphere; C) Trajectory of the intra-pathway connectivity of the social perception pathway for the right hemisphere.

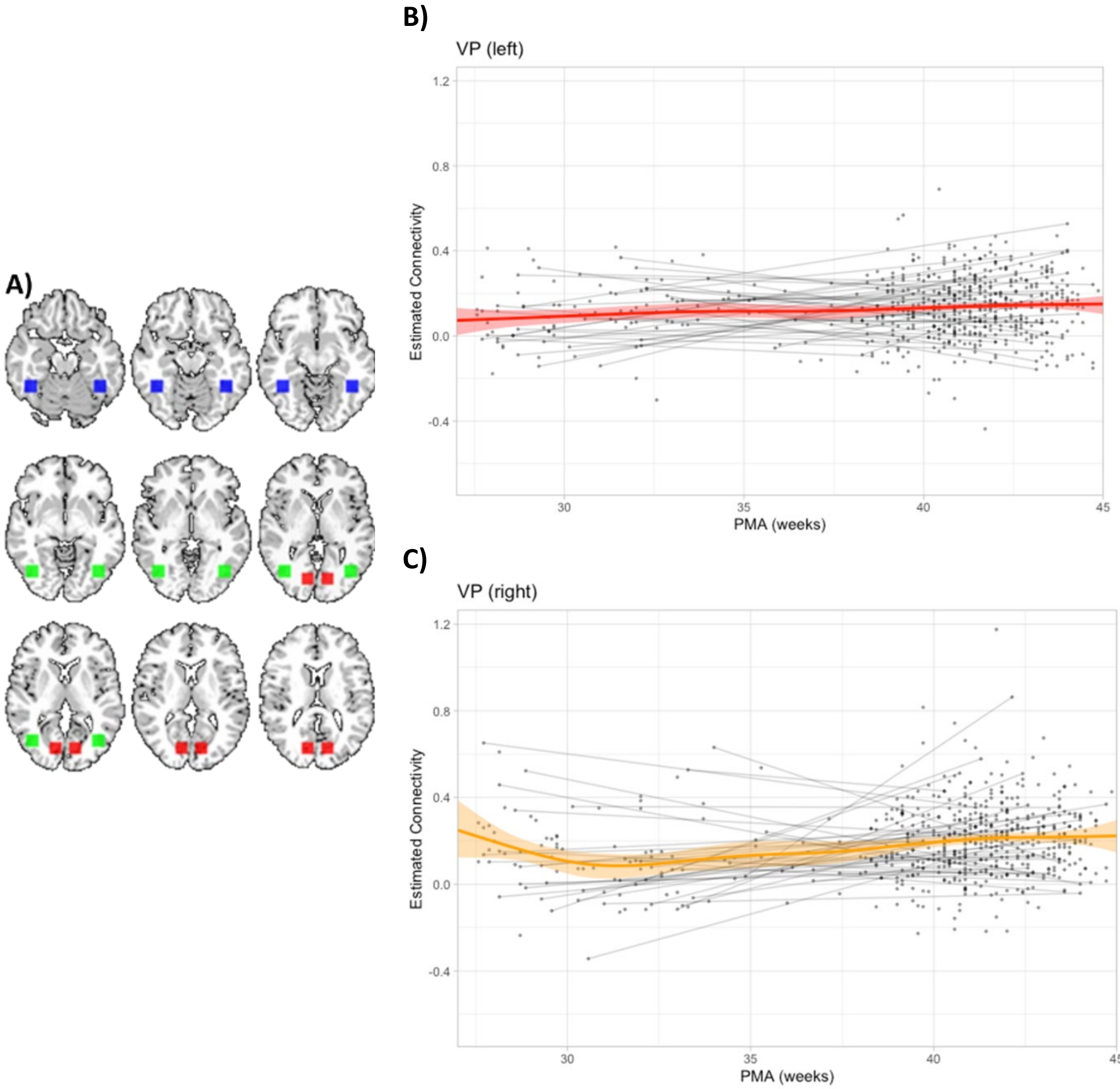

Figure S3. Longitudinal development of ventral pathway across the third trimester of gestation and perinatal transition including all data points. A) The nodes for the ventral pathway: V1 (red), lateral occipital complex (green), and fusiform gyrus (blue). B) Trajectory of the intra-network connectivity of the ventral pathway for the left hemisphere. C) Trajectory of the intra-network connectivity of the ventral pathway for the right hemisphere.
